# Correlated Decision Fusion Accompanied with Quality Information on a Multi-Band Pixel Basis for Land Cover Classification

**DOI:** 10.3390/jimaging10040091

**Published:** 2024-04-12

**Authors:** Spiros Papadopoulos, Georgia Koukiou, Vassilis Anastassopoulos

**Affiliations:** Electronics Laboratory, Physics Department, University of Patras, 26504 Patras, Greece; gkoukiou@upatras.gr (G.K.); vassilis@upatras.gr (V.A.)

**Keywords:** land cover classification, thermal infrared, PolSAR, decision fusion, majority voting, quality bit

## Abstract

Decision fusion plays a crucial role in achieving a cohesive and unified outcome by merging diverse perspectives. Within the realm of remote sensing classification, these methodologies become indispensable when synthesizing data from multiple sensors to arrive at conclusive decisions. In our study, we leverage fully Polarimetric Synthetic Aperture Radar (PolSAR) and thermal infrared data to establish distinct decisions for each pixel pertaining to its land cover classification. To enhance the classification process, we employ Pauli’s decomposition components and land surface temperature as features. This approach facilitates the extraction of local decisions for each pixel, which are subsequently integrated through majority voting to form a comprehensive global decision for each land cover type. Furthermore, we investigate the correlation between corresponding pixels in the data from each sensor, aiming to achieve pixel-level correlated decision fusion at the fusion center. Our methodology entails a thorough exploration of the employed classifiers, coupled with the mathematical foundations necessary for the fusion of correlated decisions. Quality information is integrated into the decision fusion process, ensuring a comprehensive and robust classification outcome. The novelty of the method is its simplicity in the number of features used as well as the simple way of fusing decisions.

## 1. Introduction

Technologies like remote sensing have revolutionized the way we gather information about the Earth’s surface, giving us the opportunity to monitor and classify land cover and land use in many ways. The availability of hyperspectral, Multispectral, and Synthetic Aperture Radar (SAR) and thermal infrared data has opened new possibilities for urban land cover classification, ecological land mapping, and glacier classification. The utilization of remote sensing data for land cover classification is important in dealing with various environmental and urban planning challenges. This introduction presents an overview of the research studies that have investigated the fusion of different data sources and decision-level techniques to enhance the accuracy and robustness of land cover classification.

Urban areas are dynamic environments, and monitoring land cover changes in these regions is essential for urban planning and development. Qiu et al. [1] proposed a decision-level fusion approach that leverages multi-seasonal Sentinel-2 imagery with state-of-the-art Residual convolutional neural networks (ResNet) for urban land cover classification. Their approach demonstrates superior performance in urban land cover classification by making use of multiple seasonal datasets. Xu et al. [2] proposed a novel classification approach based on multi-structure joint decision-making strategy and pretrained convolutional neural networks (CNNs) combining three different CNNs to classify land use. The study illustrates how this approach maximizes the potential of pretrained CNN structures and improves classification accuracy, especially for indistinguishable land use types. Chen et al. [3] introduced a decision-level fusion approach by combining Landsat 8 and Sentinel-1 data using decision-level fusion (DLF). Their study demonstrates that DLF enhances crop classification accuracy, showcasing the utility of data fusion in agricultural applications. Chen et al. [4] explored the complementarity of fully polarimetric SAR and optical imaging. Their approach leverages polarimetric decomposition methods and object-based decision tree classification, achieving improved accuracy by merging data from both sources. Land use classification can benefit from supervised cross-fusion methods. Rangzan et al. [5] presented a supervised cross-fusion method, combining pixel-based and supervised feature-based fusion of thermal, radar, and optical data. Their approach significantly improved classification accuracy compared to state-of-the-art fusion methods, demonstrating the effectiveness of combining multiple data sources. Machine learning classifiers have been proven effective in the hierarchical mapping of glacier surfaces. Alifu et al. [6] utilized machine learning classifiers, including k-nearest neighbors, support vector machine, gradient boosting, decision tree, random forest, and multi-layer perceptron, to classify debris-covered glaciers. Their approach demonstrated high classification accuracy, particularly when combining multiple data sources, making it suitable for precise delineation of debris-covered glaciers in various regions.

The fusion of hyperspectral and LiDAR data provides valuable insights for surface material classification. Jia et al. [7] introduced a multiple feature-based super pixel-level decision fusion (MFSuDF) method, combining kernel principal component analysis (KPCA) for dimension reduction and Gabor features for discriminative information. The study presents successful land classification results by combining information from different modules, resulting in an impressive classification accuracy. Fusing data from Sentinel-1 and Sentinel-2 satellites is pivotal for urban land cover mapping. Bui and Mucsi [8] compared two fusion methods, layer-stacking and Dempster–Shafer (D-S) theory-based approaches, at the decision level. Their results showed that D-S theory-based fusion provided the best mapping accuracy, highlighting the significance of decision-level fusion in enhancing urban land cover mapping. SAR imagery, with its polarization bands, presents unique challenges in crop discrimination. Maggiolo et al. [9] introduced a decision fusion approach for optical–SAR image classification integrated with Markov Random Fields (MRFs). Their method optimizes classification by integrating spatial-contextual information, making it suitable for large-scale applications like global climate change monitoring. Zhu et al. [10] proposed a SAR image fusion classification method based on decision-level combination of multi-band information. Their approach incorporates (D-S) evidence theory and convolutional neural networks, achieving improved classification accuracy for multi-band SAR images. Fatemeh Tabib Mahmoudi et al. [11] introduced a feature-level fusion approach that leverages both SAR texture images and Landsat 8 data. Their method improves the overall classification accuracy and Kappa coefficient, showcasing the potential for feature-level fusion in urban land cover classification.

The primary objective of this study is to combine fully polarimetric SAR data with thermal infrared images in order to examine if a quality bit transmitted along the decisions to the fusion center would significantly improve the classification accuracy. To achieve this, the first step involves registering the acquired images, allowing for proper alignment and calibration. By precisely aligning the images, we ensure a stable spatial reference for later analysis and classification. After the registration process, our efforts will focus on optimal pixel-level feature extraction to capture the distinctive features of land cover types. This feature extraction stage will involve an in-depth analysis of these data, considering the unique information provided by each sensor. Then, we are going to develop a correlated decision with quality bit method to exploit the complementary nature of these modalities, enhancing the discriminative power of the classification process.

In the subsequent sections, we delve deeper into our study, beginning with Section 2, where we outline the study area and materials utilized. Following this, in Section 3, we elaborate on the preprocessing techniques employed specifically for PolSAR data. Section 4 is dedicated to discussing the feature extraction methods applied to PolSAR data, while in Section 5, we shift our focus to feature extraction techniques we used for Landsat-8 thermal infrared imagery. Our classification methodology is explained in Section 6, followed by a presentation of decision fusion technique that we used in Section 7. Finally, in Section 8, we present our conclusions that came up from the findings of this study.

## 2. Study Area and Materials

**Figure 1:**
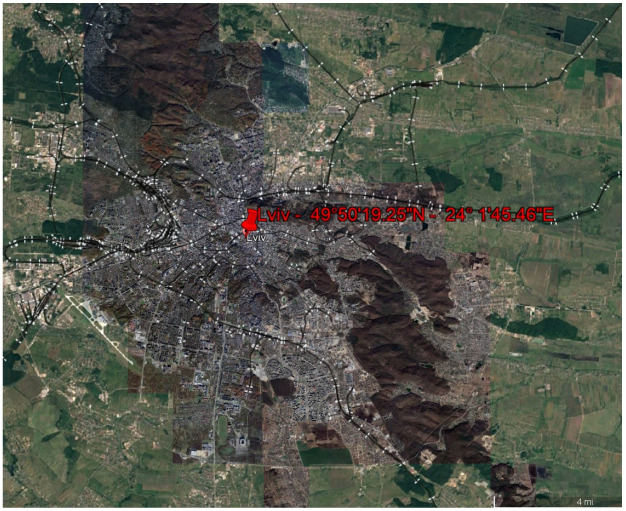
The location of the study area in Lviv, Ukraine. Map data ©2024: Google, Maxar Technologies.

A city in western Ukraine called Lviv was chosen as the study area that is located by north latitude of 49°51′ and east longitude of 24°01′. The study area consists of four main types of land cover including urban, vegetation, water and bare land. The location of the study area is depicted in Figure 1.

In our study, we used two thermal infrared bands with 100 m spatial resolution of Landsat 8 which belong to Landsat-8 OLI/TIRS-L1TP, a precision terrain product. This product includes radiometric and geometric accuracy and was acquired on 30 March 2014, at 9:14 a.m. Landsat-8 has 185 Km swath width and due to near-polar orbit, $98.2^{{}^{\circ}}$ degrees inclination, falls within the view once in every 16 days. *LST* values were calculated using RED, NIR, and 10 and 11 bands of Landsat 8 data.

Also, we used data from satellite ALOS which has absolute orbit 20,165 and has incidence angle (near–far) $24.73^{{}^{\circ}}$ and $26.53^{{}^{\circ}}$ degrees, respectively. ALOS PALSAR P1.1 Single Look Complex (SLC) product was acquired on 5 November 2009, with L band as center frequency, PLR beam mode, and 30 m spatial resolution. VV, VH, HV, and HH polarizations were used in our study in order to exploit as much information as possible. Images of Landsat 8 and ALOS PALSAR were freely downloaded from the European Space Agency or ESA (http://earth.esa.int, accessed on 1 September 2022) and Alaska Satellite facility data search (https://search.asf.alaska.edu, accessed on 1 September 2022), respectively.

## 3. Preprocessing—PolSAR

**Figure 2:**
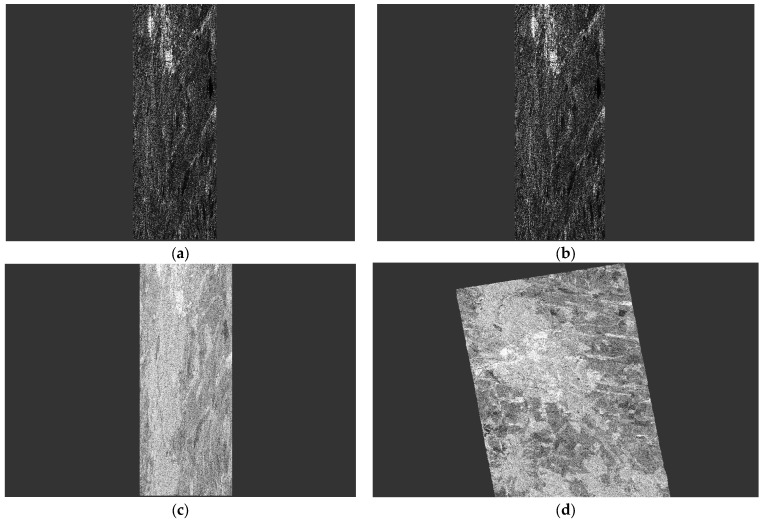
Correction of geometric distortions in the ALOS ascending image. (**a**) Amplitude of original image, (**b**) amplitude of calibrated image, (**c**) Pauli component, and (**d**) georeferenced Pauli component.

SLC PolSAR data represent raw observations (Figure 2a) that require careful preprocessing to unlock their valuable information. Employing the Sentinel Application Platform (SNAP), we engage in a methodical progression encompassing radiometric calibration [12], Pauli decomposition, and geometric Doppler terrain correction [13].

Radiometric calibration serves the pivotal purpose of translating raw digital numbers into physically meaningful units. This procedure corrects a SAR image so that the pixel values truly represent the radar backscatter of the reflecting surface but continues to maintain the geometric distortions in Figure 2b. Then, Pauli’s decomposition is used in order to transform the complex polarimetric matrices into three distinct Pauli components (see one of the components Figure 2c). This step enables a visually intuitive representation of polarimetric information, facilitating the interpretation of scattering mechanisms within the radar data. Finally, geometric Doppler terrain correction emerges as a cartographic imperative, rectifying geometric distortions attributable to variable topography. Leveraging a Digital Elevation Model (DEM) [14], this correction compensates for undulating terrain, aligning radar reflections with veracious geographic coordinates. The output is a georeferenced dataset (Figure 2d), pivotal for spatially accurate analysis and scientific interpretation.

## 4. Feature Extraction—PolSAR Data

The basic idea of the Pauli decomposition is to express the matrix [S] as the sum of elementary scattering matrices representing certain types of deterministic scattering mechanisms [15,16,17]. If we consider that the conventional orthogonal linear (h,v) basis and $S_{hv}=S_{vh}$ the Pauli basis $\left[S_{a}\right],\;\left[S_{b}\right],\;\left[S_{c}\right]$ is given by the following three 2 × 2 matrices:

(1) $$\;\;\;\left[S_{a}\right]=\frac{1}{\sqrt{2}}\left[\begin{array}{cc} 1 & 0\\ 0 & 1 \end{array}\right]$$

(2) $$\left[S_{b}\right]=\frac{1}{\sqrt{2}}\left[\begin{array}{cc} 1 & 0\\ 0 & -1 \end{array}\right]$$

(3) $$\left[S_{c}\right]=\frac{1}{\sqrt{2}}\left[\begin{array}{cc} 0 & 1\\ 1 & 0 \end{array}\right]$$

Consequently, given a measured scattering matrix [S], it can be expressed as follows:

(4) $$\left[S\right]=\left[\begin{array}{cc} S_{hh} & S_{hv}\\ S_{hv} & S_{vv} \end{array}\right]=\alpha \left[S_{a}\right]+\beta \left[S_{b}\right]+\gamma \left[S_{c}\right]$$

where

(5) $$\alpha =\frac{S_{hh}+S_{vv}}{\sqrt{2}}$$

(6) $$\beta =\frac{S_{hh}-S_{vv}}{\sqrt{2}}$$

(7) $$\gamma =\sqrt{2}S_{hv}$$

The matrix $\left[S_{a}\right]$ is the scattering matrix of a sphere, a plate, or a trihedral. In this way, the intensity of the coefficient α determines the power scattered by targets characterized by single- or odd-bounce. The second matrix, $\left[S_{b}\right]$, is the scattering mechanism of dihedral oriented at 0 degrees; consequently, β represents the power scattered by this type of targets. Finally, the third matrix $\left[S_{c}\right]$ is the scattering mechanism of a diplane oriented at 45 degrees, i.e., the coefficient γ is referred to those scatterers which are able to return to the orthogonal polarization, from which, one of the best examples is the volume scattering. All this correspondence is demonstrated in Table 1.

**Pauli color-coded representation**

The polarimetric information of the scattering matrix can be represented by the combination of intensities (${\left|S_{hh}\right|}^{2},{\left|S_{vv}\right|}^{2},2{\left|S_{hv}\right|}^{2}$) in a single RGB image. However, the main drawback is the physical interpretation of the resulting image in terms of ${\left|S_{hh}\right|}^{2},{\left|S_{vv}\right|}^{2},2{\left|S_{hv}\right|}^{2}$. Consequently, an RGB image can be formed with the intensities ${\left|\alpha \right|}^{2},{\left|\beta \right|}^{2},{\left|\gamma \right|}^{2}$, which corresponds to clear physical scattering mechanisms as shown in Table 1. The most employed codification corresponds to the following:

(8) $${\left|\beta \right|}^{2}\to red\;\;\;\;{\left|\gamma \right|}^{2}\to green\;\;{\left|\alpha \right|}^{2}\to blue$$

**Figure 3:**
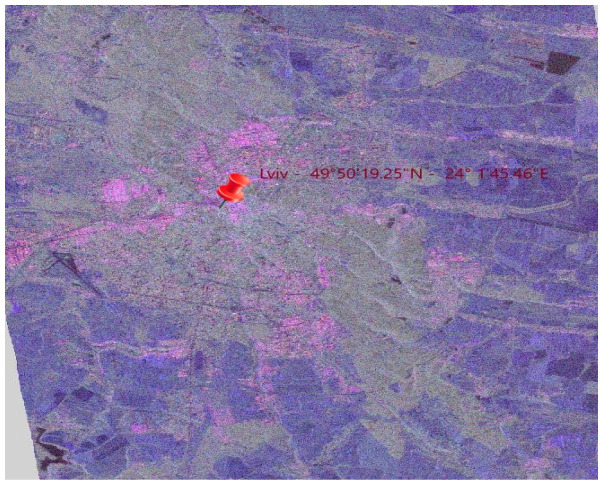
Color-coded representation of our study area from SAR data.

Incorporating the theoretical foundation elucidated earlier, we employed Pauli scattering components retrieved from SNAP software (SNAP v9.0.0), denoted as *α*, *β*, and *γ*, representing the intensities of the scattering coefficients. These values were expressed in decibels. Given that negative decibel values are incompatible with color representation, a normalization procedure was implemented for each component. This involved manipulating their histograms to scale the values within the range of 0 to 255. So, we came to the following result as shown below in Figure 3.

## 5. Feature Extraction—Landsat-8 Thermal Infrared

Streamlining the identification of specific regions [19] is facilitated by leveraging previously recorded and registered surface temperature data across diverse land cover types. This meticulous characterization enables the determination of the most likely land cover class and an evaluation of its practical significance. In the estimation of land surface temperature (*LST*), we utilized raw data from a single-date Landsat-8 remote sensing imagery [20,21,22], specifically incorporating band 10 and 11, which are significantly influenced by stray light, for specific procedures. Various methods are available for estimating and calculating *LST* [23,24,25], including Split-Window (SW), Dual-Angle (DA), and Single-Channel (SC) algorithms. In this research, we opted for the Split-Window approach, integrating thermal band 10 [26] and Normalized Difference Vegetation Index (*NDVI*) data obtained for the study area.

**Figure 4:**
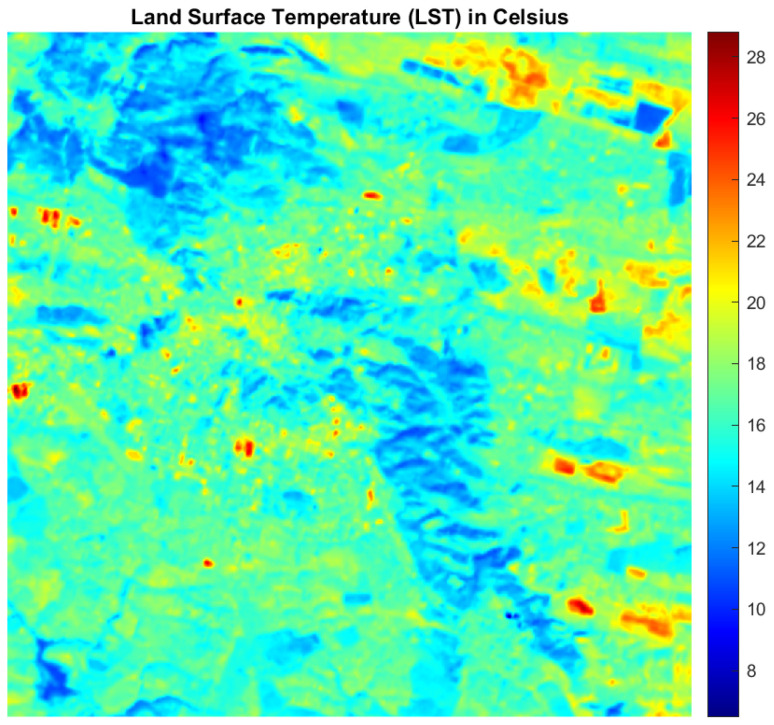
Heat map of study area using *LST* equation for band 10.

The heat map of Figure 4 resulted from the equation for calculating *LST* which is articulated as follows:

(9) $$LST=\frac{BT}{\left[1+\left\{\left(\frac{\lambda_{BT}}{\rho }\right)\ln\varepsilon \lambda \right\}\right]}$$

where BT denotes brightness temperature, $\lambda_{BT}$ represents the wavelength of the band, ελ/LSE signifies land surface emissivity, and ρ is equivalent to 1438. Brightness temperature refers to the temperature of a blackbody corresponding to the radiance detected by a sensor. According to NASA (2012) [27], it is the temperature measured by the satellite at the moment the image was captured and does not directly mirror the actual temperature of the bare land. Instead, it mirrors the temperature at the satellite location [28,29]. To transform the thermal infrared sensor (TIRS) bands data from spectral radiance to brightness temperature, the thermal constants provided in the metadata file are utilized. The equation employed for this conversion is recognized as the brightness temperature (10).

(10) $$BT=\frac{K_{2}}{\ln\left[\left(\frac{K_{1}}{L_{\lambda }}\right)+1\right]}-273.15$$

where $K_{1}$ and $K_{2}$ denote the thermal zone-specific thermal constants using only zone 10. Landsat-8 offers basic constants, including thermal constants and rescaling factors, for *LST* estimation, which are all available in the metadata file of Landsat satellite images. $L_{\lambda }$ represents the Top of Atmospheric spectral radiance (TOA). To determine the brightness temperature (*BT*), Equation (11) is applied, using the Top of Atmospheric spectral radiance (TOA).

(11) $$L_{\lambda }=M_{L}Qcal+A_{L}$$

The *M_L_* factor, denoted by the variable (radiance_mult_band_10), signifies the band-specific multiplicative rescaling factor, while the $A_{L}$ factor, represented by (radiance_add_band_10), signifies the band-specific additive rescaling factor for the Qcal of band 10 image.

The Normalized Difference Vegetation Index (*NDVI*), derived from satellite data, is intricately connected to drought conditions. The assessment of green density on a land patch involves observing the distinct colors (wavelengths) of visible and near-infrared sunlight reflected by plants, with band 4 and band 5 (red and near-infrared bands, respectively) utilized for calculating the Normal *NDVI*. Estimating *NDVI* is crucial due to its correlation with vegetation abundance and providing insights into the general vegetation conditions. Subsequent to *NDVI* calculation, the proportion of vegetation (*P_V_*) needs assessment, which is closely linked with *NDVI*, and emissivity (ε) calculation becomes pivotal, as emissivity is related to *P_V_*.

(12) $$NDVI=\frac{NIR(band5)-R(band4)}{NIR(band5)+R(band4)}$$

To calculate the blackbody radiation resulting from the bare land surface temperature, the Earth’s surface emissivity is used. Several approaches are used to estimate the emissivity at the Earth’s surface, Equation (13) being one of them. One method incorporates NDVI, taking into account the vegetation ratio ($P_{V}$) to determine the Earth’s surface temperature in Celsius.

For this purpose, the equation for calculating the emissivity (*ε*) of the Earth’s surface is used, as presented in Equation (13).

(13) $$\varepsilon =\varepsilon_{V\lambda }P_{V}+\varepsilon_{S\lambda }\left(1-P_{V}\right)+C_{\lambda }$$

(14) $$P_{V}={\left(\frac{NDVI-{NDVI}_{S}}{{NDVI}_{V}-{NDVI}_{s}}\right)}^{2}$$

where ε = land surface emissivity, $\varepsilon_{S\lambda }$ = soil emissivity, $\varepsilon_{V\lambda }$ = vegetation emissivity, $P_{V}$ = proportion of vegetation, and $C_{\lambda }$ = surface roughness taken as a constant value of 0.009.

## 6. Classification

### 6.1. Registration

**Figure 5:**
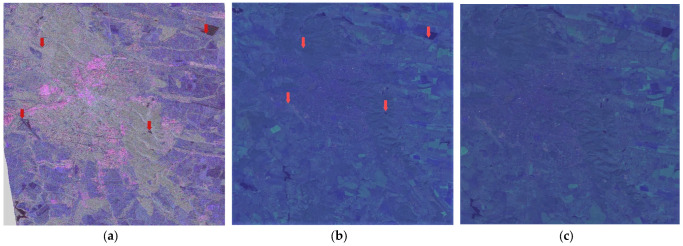
Registration with affine transformation between SAR and thermal infrared data. (**a**) Fixed SAR image, (**b**) moving thermal infrared image, and (**c**) registered thermal infrared image.

As it is known, thermal IR data from Landsat-8 and SAR data from ALOS have different spatial resolutions. So, after preprocessing, we need to apply a transformation to eliminate possible translation, rotation, and scaling distortions between the two data to be able to assign to the pixels the characteristics from the bands we have used. Registration was performed based on mutual information. An affine transformation was performed on each data after correlating the regions of overlap in the images. The registration procedure was performed with the aid of MATLAB’s “cpselect” toolbox [30]. Using “cpselect”, the regions of mutual information were manually selected and were fed into the algorithm in the form of four control points as depicted in Figure 5a,b. The transformation factors calculated from the RGB SAR image were then applied to all layers in the thermal infrared. As a result, we have the registered thermal infrared image as depicted in Figure 5c.

### 6.2. Sensor Training

The scenario employed in this work was to investigate the performance of the five-feature vector, i.e., the three Pauli’s coefficients and the two temperature indicators. Four different areas are used for examining the behavior of the vector namely: water, forest, urban and bare land. The statistics of the five-feature vector for each type of land cover is studied by considering four windows of 21×21 pixels, with one of each land cover type as training dataset. Each pixel is represented by a vector of five values, with the intensities of scattering coefficients *α*, *β*, and *γ* and two land surface temperatures T_1_ and T_2_ coming from the two thermal infrared bands. In Table 2, the median value and the standard deviation of the five values of the feature land type are presented.

### 6.3. Classification

To perform the classification process for a random pixel, we checked where the feature vector values are by comparing it to each histogram of the corresponding feature for each land cover type.

**Figure 6:**
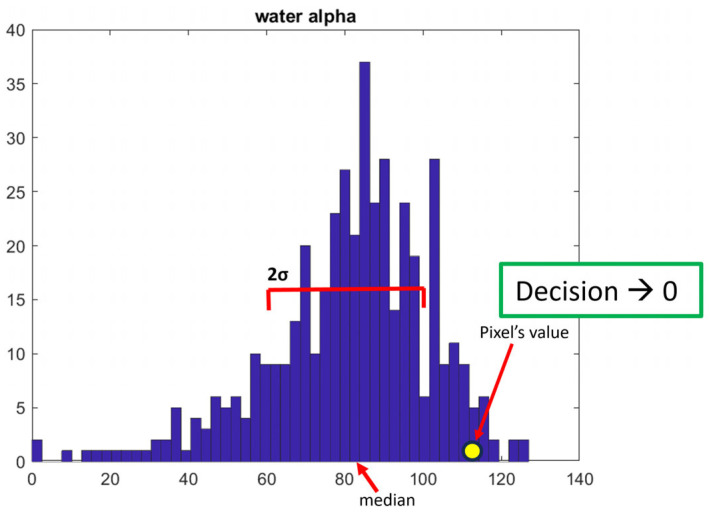
Decision rule used for classification.

Each of the decisions is obtained based on the location of the specific value of the vector component on the corresponding histogram. An example is given in Figure 6 where the histogram of Pauli coefficient *α* is given, along with the position of its median, the width of the standard deviation, and the position of the corresponding Pauli coefficient value of the unknown pixel. In the demonstrated case the decision is 0. The decision is one only when the corresponding coefficient lies between -std and std around the median.

For example, a water pixel based on the process described above gave the results of Table 3. As we expected, after comparing this particular pixel with the statistics we have extracted, we obtained more ones (presence) for the land cover type corresponding to water.

## 7. Decision Fusion

As previously mentioned, we utilized the aforementioned windows as training data to establish value ranges for categorizing random pixels into water, urban, forest, and bare land categories. Our approach involved employing majority voting to determine the predominant class within a region (Table 4).

In the evaluation of our method, we randomly tested 576 pixels, with 144 pixels sampled from each area. The results revealed 396 correct classifications, translating to a percentage accuracy of 68.8%. Specifically, our method achieved 94% accuracy in classifying urban areas, 75% in water types, 56% in forest types, and encountered the highest error rate of 50% in accurately classifying bare land areas.

**Figure 7:**
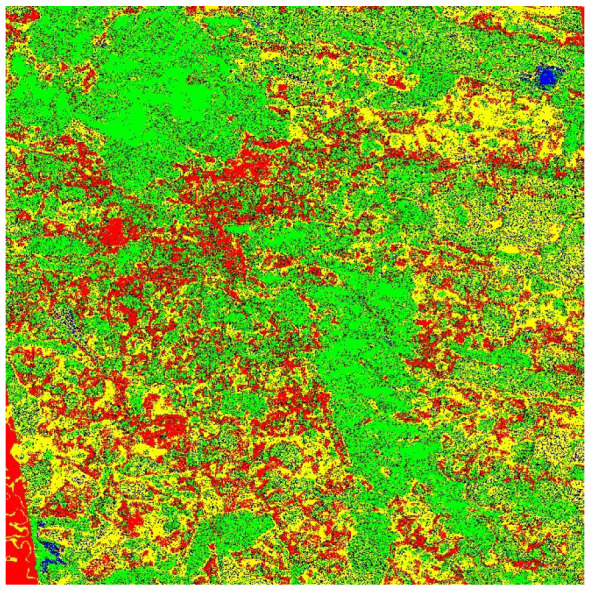
Color map of the city of Lviv. Red: urban pixels, yellow: bare land, green: forest, and Blue: water.

Although the results achieved through majority voting were satisfactory, we hypothesized that incorporating a quality bit could enhance the decision-making process. This addition not only helped in clarifying cases where uncertainty existed in the decision, such as instances where an equal number of votes were received for multiple land cover types, but also served to reinforce clear decisions. After implementing the quality bit, our assumptions were validated. We observed a substantial improvement in overall accuracy, with an increase of 10.2%. Notably, accuracy improvements were evident in 4 out of the 4 land cover types, specifically a 1% increase in urban types, a 2% improvement in water types, a 19% enhancement in forest types, and a 19% boost in bare land types—addressing significant weaknesses in our coverage classification. Taking into consideration the results, we created a colormap of the study are with the classified pixels (Figure 7).

### Discussion on Fusion Results

Going through this process of classification and decision fusion, in addition to the knowledge we gained, we also understood the pieces that we could investigate in the future to reach a more satisfactory accuracy. The kind of decomposition that could be used in a future publication should achieve better separation in the scattering coefficients. Additionally, the discovery and implementation of more characteristics among the pixels will help us better distinguish areas of mixed land cover, and a lot of that will contribute to our goal.

Looking ahead, the field presents challenges and opportunities. Addressing the open challenges in this research domain, such as refining the integration of quality information for decision fusion, remains a key focus. The dynamic nature of land cover, environmental changes, and the ever-increasing volume of data pose ongoing challenges that require the continuous adaptation of our methodologies.

On the flip side, these challenges also bring forth opportunities for innovation. Advances in machine learning algorithms, sensor technologies, and computational capabilities open doors for more sophisticated and accurate classification methods. Exploring synergies with emerging technologies like remote sensing and artificial intelligence could unlock new possibilities for enhanced land cover analysis.

In summary, the future prospect for this research domain involves navigating challenges while seizing opportunities for advancements. Continuous exploration, adaptation, and the integration of cutting-edge technologies will play pivotal roles in shaping the trajectory of our research and contributing to a more comprehensive understanding of land cover dynamics.

## 8. Conclusions

In this research, we proposed a novel approach for land cover classification by integrating fully polarimetric Synthetic Aperture Radar (SAR) and thermal infrared data. Our methodology involves a pixel-level correlated decision fusion, which enhances the accuracy and robustness of land cover classification. Ιn reference [31], a multitude of decomposition methods are analyzed and presented that have been used to extract the biophysical scattering behavior of SAR data. However, in this work we utilized Pauli’s decomposition components and land surface temperature (*LST*) as features to extract local decisions for each pixel, considering the unique information provided by each sensor.

Our study area, located in Lviv, Ukraine, consists of four main land cover types: urban, vegetation, water, and bare land. We employed data from Landsat 8 and ALOS satellites, combining thermal infrared and fully polarimetric SAR data to achieve a better understanding of land cover characteristics. The preprocessing steps involved radiometric calibration, Pauli’s decomposition, and geometric Doppler terrain correction for SAR data. Feature extraction included the calculation of scattering coefficients from fully polarimetric SAR data and the estimation of land surface temperature (*LST*) from Landsat thermal infrared data.

To assess the correlation, we analyzed truth tables such as Table 3 and their covariance matrices, we selected four random pixels from each land cover type, and we formed two pairs: one comprising two successful classifications and another with one successful and one failed classification. The truth tables, such as those illustrated in Table 5, were constructed to represent the pixel decisions for each pair. The tables include values of 0 or 1 corresponding to specific features. We used these truth tables later to calculate covariance matrices for each pair, revealing the relationships between pixel decisions. The results, presented in the final covariance matrix table (Table 6), demonstrate the covariance values between pairs of pixel decisions for water, urban, forest, and bare land. The positive and negative values in the matrices indicate the strength and direction [32] of the correlation between the selected pixel classifications. We observed that most decisions have a strong correlation without being important if we compare two successful or one successful and one failed pixel classification.

This extensive approach provides a comprehensive insight into the interdependence of pixel decisions across various land cover types, offering valuable information for understanding the performance and reliability of the classification model.

Furthermore, the classification and decision fusion process utilized a window-based training dataset for each land cover type. The highest accuracy was accomplished in urban areas (94%), followed by water (75%), forest (56%), and bare land (50%). Decision fusion was achieved through majority voting, and the method demonstrated an overall accuracy of 68.8%. In conclusion, our proposed methodology of correlated decision fusion accompanied with a quality bit was proved to be effective in enhancing the accuracy of land cover classification by **10.2%**. The integration of fully polarimetric SAR and thermal infrared data provides complementary information, and the pixel-level fusion approach ensures a comprehensive understanding of diverse land cover types in the study area.

## Figures and Tables

**Table 1:** Pauli bases and the corresponding meanings [18].

Pauli Basis	Meaning
$S_{a}$	**Single- or odd-bounce scattering:** This occurs when a radar signal interacts with a target and undergoes a single reflection or bounce before reaching the radar sensor.
$S_{b}$	**Double- or even-bounce scattering:** This can happen, for instance, when radar waves hit a surface, reflect off, and then reflect again off another surface before returning to the sensor.
$S_{c}$	**Volume scattering:** This type of scattering is more complex and involves multiple interactions within the target volume, leading to a scattering signal that does not follow a simple direct path (forest canopy).

**Table 2:** Median and standard deviation values of histograms of the test windows.

	Water Test		Forest Test		Urban Test		Bare Land Test	
	Median	Std	Median	Std	Median	Std	Median	Std
***α***	84	20.84	151	23.64	230	28.22	144	24.58
***β***	78	22.96	139	23.16	211	28.85	95	22.04
***γ***	59	21.81	137	22.00	140	27.16	76	23.88
**Τ_1_**	11.20	0.1	11.69	1.21	16.79	0.53	16.24	0.43
**Τ_2_**	10.98	0.12	11.69	1.21	16.79	0.48	15.92	0.43

**Table 3:** Truth table of a correct classified random water pixel.

Water					
	*α*	*β*	*γ*	Τ_1_	Τ_2_
**Water**	1	1	1	1	1
**Urban**	0	0	0	0	0
**Forest**	0	0	0	1	1
**Bare land**	0	1	1	0	0

**Table 4:** This table illustrates truth tables indicating the existence (1) or non-existence (0) of pixels linked to each land cover type. The determination of presence (1) is contingent upon the coefficient value falling within the range established by the median plus or minus the standard deviation.

Water1					
Pixel	*α*	*β*	*γ*	Τ_1_	Τ_2_
**Water**	**1 (0)**	**1 (1)**	**1 (0)**	**1 (1)**	**1 (1)**
Urban	0	0	0	0	0
Forest	0	0	0	1 (1)	1 (0)
Bare land	0	1 (0)	1 (1)	0	0
**Water2**					
**Pixel**	***α***	***β***	***γ***	**Τ_1_**	**Τ_2_**
**Water**	**1 (1)**	**1 (1)**	**1 (0)**	**0**	**0**
Urban	0	0	0	0	0
Forest	0	0	0	0	1 (1)
Bare land	0	1 (0)	1 (1)	0	0
**Forest1**					
**Pixel**	***α***	***β***	***γ***	**Τ_1_**	**Τ_2_**
Water	0	0	0	0	0
Urban	0	0	1 (0)	0	0
**Forest**	**0**	**1 (1)**	**1 (0)**	**0**	**0**
Bare land	0	0	0	0	0
**Forest2**					
**Pixel**	***α***	***β***	***γ***	**Τ_1_**	**Τ_2_**
Water	0	0	0	0	0
Urban	0	0	1 (0)	0	0
**Forest**	**0**	**1 (0)**	**0**	**1 (0)**	**1 (0)**
Bare land	0	0	0	0	0
**Urban1**					
**Pixel**	***α***	***β***	***γ***	**Τ_1_**	**Τ_2_**
Water	0	0	0	0	0
**Urban**	**1 (0)**	**1 (0)**	**1 (1)**	**0**	**1 (1)**
Forest	0	0	0	0	0
Bare land	0	0	0	0	0
**Urban2**					
**Pixel**	***α***	***β***	***γ***	**Τ_1_**	**Τ_2_**
Water	0	0	1 (1)	0	0
**Urban**	**1 (0)**	**1 (1)**	**0**	**1 (1)**	**1 (1)**
Forest	0	0	0	0	0
Bare land	0	0	1	0	0
**Bare land 1**					
**Pixel**	***α***	***β***	***γ***	**Τ_1_**	**Τ_2_**
**Water**	**0**	**0**	**1 (1)**	**0**	**0**
**Urban**	**0**	**0**	**0**	**1 (0)**	**1 (0)**
**Forest**	**1 (1)**	**1 (0)**	**0**	**0**	**0**
**Bare land**	**1 (0)**	**0**	**1 (0)**	**0**	**0**
**Ground2**					
**Pixel**	***α***	***β***	***γ***	**Τ_1_**	**Τ_2_**
Water	0	0	0	0	0
Urban	0	0	0	0	0
Forest	1 (1)	0	0	0	0
**Bare land**	**1 (1)**	**1 (0)**	**1 (0)**	**0**	**0**

**Table 5:** Pairs of truth tables comparing two successful classified pixels and one successful and one failed from each land cover type, including quality bit in parenthesis, which were used for the calculation of the covariance matrix and furthermore, the investigation of correlation between decisions.

Water										
	Successful					Successful				
	***α***	***β***	***γ***	**Τ_1_**	**Τ_2_**	***α***	***β***	***γ***	**Τ_1_**	**Τ_2_**
**Water**	1 (0)	1 (1)	1 (0)	1 (0)	1 (1)	1 (1)	1 (1)	1 (0)	0	0
**Urban**	0	0	0	0	0	0	0	0	0	0
**Forest**	0	0	0	1 (1)	1 (0)	0	0	0	0	1 (1)
**Bare land**	0	1 (0)	1 (1)	0	0	0	1 (0)	1 (1)	0	0
	**Successful**					**Failed**				
**Water**	1 (1)	1 (0)	1 (1)	0	0	1 (1)	0	1 (1)	0	0
**Urban**	0	0	0	0	0	0	0	0	0	0
**Forest**	0	0	0	1 (1)	1 (1)	0	0	0	1 (0)	1 (1)
**Bare land**	0	1 (1)	0	0	0	0	1 (0)	1 (1)	0	0
**Urban**										
	**Successful**					**Successful**				
	***α***	***β***	***γ***	**Τ_1_**	**Τ_2_**	***α***	***β***	***γ***	**Τ_1_**	**Τ_2_**
**Water**	0	0	0	0	0	0	0	1 (1)	0	0
**Urban**	1 (1)	1 (0)	1 (1)	0	1 (1)	1 (0)	1 (1)	0	1 (1)	1 (1)
**Forest**	0	0	0	0	0	0	0	0	0	0
**Bare land**	0	0	0	0	0	0	0	1 (0)	0	0
	**Successful**					**Failed**				
**Water**	0	0	0	0	0	0	0	1 (1)	0	0
**Urban**	0	1 (1)	1 (1)	1 (0)	1 (0)	0	1 (0)	0	0	0
**Forest**	0	0	1 (1)	0	0	1 (0)	0	0	0	0
**Bare land**	0	0	0	1 (1)	0	1 (0)	0	1 (0)	0	0
**Forest**										
	**Successful**					**Successful**				
	***α***	***β***	***γ***	**Τ_1_**	**Τ_2_**	***α***	***β***	***γ***	**Τ_1_**	**Τ_2_**
**Water**	0	0	0	0	0	0	0	0	0	0
**Urban**	0	0	1 (1)	0	0	0	0	0	0	0
**Forest**	1 (1)	1 (0)	1 (1)	1 (0)	1 (0)	1 (0)	1 (1)	0	1 (0)	1 (0)
**Bare land**	1 (1)	0	0	0	0	0	0	1 (1)	0	0
	**Successful**					**Failed**				
**Water**	0	0	0	0	0	0	0	0	0	0
**Urban**	0	0	1 (1)	0	0	0	0	1 (0)	1 (0)	0
**Forest**	1 (1)	1 (1)	1 (0)	0	0	1 (0)	1 (0)	1 (0)	0	0
**Bare land**	1 (0)	0	0	0	0	1 (1)	0	0	1 (1)	1 (0)
**Bare land**										
	**Successful**					**Successful**				
	***α***	***β***	***γ***	**Τ_1_**	**Τ_2_**	***α***	***β***	***γ***	**Τ_1_**	**Τ_2_**
**Water**	0	1 (1)	1 (0)	0	0	0	0	0	0	0
**Urban**	0	0	0	0	0	0	0	1 (0)	1 (1)	1 (0)
**Forest**	1 ()	0	0	0	0	1 (1)	0	0	0	0
**Bare land**	1 (1)	1 (0)	1 (1)	0	0	1 (1)	1 (0)	0	1 (0)	1 (0)
	**Successful**					**Failed**				
**Water**	0	0	0	0	0	0	0	1 (1)	0	0
**Urban**	0	0	0	0	0	0	0	0	1 (0)	1 (0)
**Forest**	1 (1)	0	0	0	0	1 (1)	1 (0)	0	0	0
**Bare land**	1 (1)	1 (0)	1 (0)	0	0	1 (0)	0 (0)	1 (0)	0	0

**Table 6:** Calculated covariance matrices of two pairs of pixels for each land cover type.

Covariance Matrices								
	Water		Urban		Forest		Bare Land	
**Successful–Successful**	0.2605	0.1447	0.1684	0.0947	0.2395	0.1184	0.2211	0.0316
	0.1447	0.1974	0.0947	0.2211	0.1184	0.1974	0.0316	0.2526
**Successful–Failed**	0.2211	0.1684	0.2211	−0.0263	0.1974	0.1579	0.1684	0.0842
	0.1684	0.2211	−0.0263	0.1974	0.1579	0.2526	0.0842	0.2395

## Data Availability

Landsat-8 imagery downloaded from ESA Landsat Online Catalogue (https://landsat-diss.eo.esa.int/socat/LANDSAT-8_L1/, accessed on 1 September 2022) it is a Landsat-8 Collection 2 Level 1 product. PolSAR data downloaded from ASF Data Search Vertex (https://search.asf.alaska.edu/#/, accessed on 1 September 2022) with product name ALPSRP201560990-L1.1.
